# Anti-Anemic Effect of Antioxidant-Rich Apple Vinegar against Phenylhydrazine-Induced Hemolytic Anemia in Rats

**DOI:** 10.3390/life12020239

**Published:** 2022-02-04

**Authors:** Driss Ousaaid, Asmae El Ghouizi, Hassan Laaroussi, Meryem Bakour, Hamza Mechchate, Imane Es-safi, Omkulthom Al Kamaly, Asmaa Saleh, Raffaele Conte, Badiaa Lyoussi, Ilham El Arabi

**Affiliations:** 1 Laboratory of Natural Substances, Pharmacology, Environment, Modeling, Health and Quality of Life, Faculty of Sciences Dhar El Mahraz, University Sidi Mohamed Ben Abdellah, Fez P.O. Box 3000, Morocco; driss.ousaaid@usmba.ac.ma (D.O.); asmae.elghouizi@usmba.ac.ma (A.E.G.); hassan.laaroussi@usmba.ac.ma (H.L.); meryem.bakour@usmba.ac.ma (M.B.); lyoussi@gmail.com (B.L.); Ilham.elarabi@gmail.com (I.E.A.); 2 Laboratory of Inorganic Chemistry, Department of Chemistry, University of Helsinki, P.O. Box 55, FI-00014 Helsinki, Finland; Imane.essafi1@usmba.ac.ma; 3 Department of Pharmaceutical Sciences, College of Pharmacy, Princess Nourah bint Abdulrahman University, P.O. Box 84428, Riyadh 11564, Saudi Arabia; ASAli@pnu.edu.sa; 4 Research Institute on Terrestrial Ecosystems (IRET)—CNR, Via Pietro Castellino 111, 80131 Naples, Italy; Raffaele.conte86@tiscali.it

**Keywords:** natural products, apple vinegar, anemia, phenylhydrazine, antioxidant activity, LC-MS/MS

## Abstract

This study aims to examine the ability of apple vinegar on phenylhydrazine (PHZ)-induced hemolytic anemia in Wistar rats. In vitro, phenolic and flavonoid content and antioxidant activity were determined. In vivo, phenylhydrazine (10 mg/kg) was injected intravenously into rats for 4 days and then treated with apple vinegar daily by gavage (1 mL/kg) for five weeks. high level of polyphenols and flavonoids (90 ± 1.66 mg GAE/100 mL and 7.29 ± 0.23 mg QE/100 mL, respectively) were found in the apple vinegar which gives it a good ability to scavenge free radicals (TAC = 4.22 ± 0.18 mg AAE/100 mL and DPPH, IC_50_ = 0.49 ± 0.004 µL/ml). The phytochemical composition of apple vinegar revealed the presence of numerous bioactive compounds including arbutin, apigenin, sinapic, ferulic and trans-ferulic acids. The major antioxidant components in apple vinegar were ferulic and trans-ferulic acids (40% and 43%, respectively). PHZ treatment induced changes in platelets, blood cell count, mean corpuscular volume, hemoglobin concentration and mean capsulated hemoglobin. However, the co-administration of apple vinegar revealed its capacity to ameliorate the changes induced by phenylhydrazine. Therefore, apple vinegar use could have a positive impact on the prevention of hemolytic anemia induced by phenylhydrazine due to the antioxidant properties of its major components.

## 1. Introduction

Blood is a liquid tissue that circulates in our body through blood vessels. The diminution of the number of erythrocytes below the normal range leads to the appearance of anemia [1]. Multiple factors increase the destruction rate of erythrocytes, including infections, drugs and hemoglobinopathies, which decrease the capacity of blood to carry oxygen [2]. The diminution of the quality or quantity of erythrocytes can be translated to the onset of anemia. The incidence of drug-induced hemolytic anemia is rare, and it is estimated at 1 per million people [3]. It has been demonstrated by sufficient evidence that about 130 drugs can induce hemolytic anemia via numerous mechanisms [4]. Depending on the site of hemolysis, drug-induced hemolytic anemia can be classified as intravascular and extravascular. Intravascular hemolysis occurs when blood contains exogenous toxic agents or due to complement fixation with red blood cells (RBCs). On the other hand, extravascular hemolysis appears in the spleen and the liver and results from the phagocytosis of abnormal RBCs [5]. It has been reported that dimethyl fumarate, amoxicillin-clavulanate, ceftriaxone, flurbiprofen and vancomycin induced hemolytic anemia [4,6,7,8,9]. One drug with a toxic effect on red blood cells is phenylhydrazine (PHZ), which could be useful for the treatment of polycythemia vera and fever [10]. However, its negative effect on red blood cells limits its medicinal use [10]. Due to PHZ’s activation of reactive oxygen species production, it has been linked to oxidative stress [10]. It has been shown that oxidative stress is involved in the aging and apoptosis of erythrocytes, thus inducing hemolysis [11,12,13]. Oxidative stress controls numerous physiological cell functions, including proteasome function, immune response, platelet generation, mitochondrial function and reproductive cell function [14,15,16,17], whilst the exacerbation of its production induces oxidation of nucleic acid, proteins and lipids, leading to cell destruction [11].

In recent years, natural products (NPs) have attracted the attention of the scientific community, and this interest is only growing [18]. Many different biological activities and drug-like properties are the consequence of the unique chemical diversity that has arisen in natural products over millions of years [19]. Since the dawn of time, herbal remedies have relied heavily on the use of natural products as active ingredients [20,21,22]. Due to economic considerations, personal convictions, or the difficulty of obtaining pharmaceutical goods, many people throughout the world still turn to age-old medical systems like Ayurveda, Chinese medicine, and African herbal medicines when seeking treatment [18]. Modern pharmacology relies heavily on natural products for the development of new lead molecules and scaffolds [23,24,25,26]. A large number of natural products are widely used in folk medicine to prevent and/or to alleviate anemia, including herbs and bee products [27,28,29]. Additionally, fruit by-products such as apple vinegar have been documented as having several biological properties due to its rich composition in phenolic compounds [30,31,32,33,34,35,36,37,38]. Apple vinegar is considered to be an antioxidant, antihyperglyceamic, antihyperlipidimic and hepato-nephroprotective agent [39,40,41,42]. Apple vinegar contains a large group of bioactive compounds with high antioxidant potential, which counteract reactive oxygen species (ROS) [43]. Previously, it has been shown that the administration of apple vinegar attenuated the disorder and histological damage induced by nicotine in rat’s liver, normalized hepatic enzymes and enhanced the antioxidant defense system [32,42]. The advantageous properties of apple vinegar might be ascribed to its wealth of bioactive ingredients. Unfortunately, little attention has been paid to the evaluation of the ability of apple vinegar to prevent phenylhydrazine-induced hemolytic anemia.

There is no documented scientific report on the use of apple vinegar in the treatment or prevention of hemolytic anemia. Within this frame, the current work was designed to scientifically seek the efficiency of apple vinegar on phenylhydrazine-induced hemolytic anemia in albino rats.

## 2. Materials and Methods

### 2.1. Sample of Vinegar

Apple vinegar (*Malus domestica* Borkh, *Golden delicious* variety) was purchased in the Midelt area (32°40′48″ N, 4°43′59″ W) in Morocco. The vinegar sample was stored in the fridge (4°) until the experiment analysis.

### 2.2. Quantitative Analysis of Phytochemicals

The quantification of phenolic content was determined using colorimetric methods as described previously by [44]. Briefly, 50 µL of apple vinegar was mixed with 0.5 mL of Folin–Ciocalteu reagent solution (0.2 N), which had been previously prepared. Sodium carbonate solution (400 µL) was added to the mixture. The absorbance of the mixture was read at 760 nm after 2 h of incubation. The quantification of flavonoids content was determined according to the modified method from [45]. Briefly, 150 µL of sodium nitrite solution (5%) was mixed with 50 µL of sample, 150 µL of aluminum trichloride solution (10%) and 300 µL of sodium acetate (1%). The absorbance was read at 510 nm. Results were expressed as mg equivalent of gallic acid per one milliliter of vinegar for TPC and mg equivalent of quercetin per one milliliter of vinegar for TFC.

### 2.3. Antioxidant Analysis

The total antioxidant capacity of apple vinegar was determined according to the modified method from [46]. Briefly, 25 µL of sample was mixed with 1mL of phosphomolybdenum. In complete darkness, the mixture was incubated at 95 °C in a water bath. In the next step, the absorbance was measured at 695 nanometers.

The free radical scavenging activity was determined according to the modified method from [47]. First, 25 µL of sample was mixed with 1 mL of DPPH solution (150 µM) prepared in ethanol with an absorbance of 0.700 ± 0.01. The absorbance was read at 517 nm after incubating the mixture for 1 h in the dark at room temperature. The determination of the percentage inhibition (PI) of DPPH radical was determined based on the percentage inhibition of free radical DPPH using the following formula:

(1) $$\mathrm{PI}\left(\%\right)=\left(\frac{\mathrm{controle}\;\mathrm{absorbance}-\mathrm{sample}\;\mathrm{absorbance}\;}{\mathrm{controle}\;\mathrm{absorbance}\;}\right)\times 100$$

### 2.4. Mineral Composition

The analysis of mineral elements was determined using ICP-AES, as previously described by [48]. One milliliter of sample was treated with 10mL of nitric acid and filtered, and then the mixture was made up to 50 mL with distilled water.

### 2.5. LC-MS/MS Analysis of the Polyphenolic Compounds of Apple Vinegar

80 mg of apple vinegar sample was treated with 1 mL of ethanol then the mixture was sonicated during 60 min at 45°C. A Shimadzu Ultra-High-Performance Liquid Chromatograph (Nexera XR LC 40) combined with an MS/MS detector (LCMS 8060, Shimadzu Italy, Milan, Italy) was used to determine phenolic profile of our sample. The apparatus was fitted as follows: nebulizing gas flow: 2.9 L/min, heating gas flow: 10 L/min, interface temperature: 300 °C, DL temperature: 250 °C, heat block temperature: 400 °C, and drying gas flow: 10 L/min. One solvent was used as mobile phase, acetonitrile: water + 0.01% formic acid (5:95, *v/v*). The identification of the molecules was confirmed by comparing the typical fragment identified with those in our in-house developed library of molecules (see Supplementary File).

### 2.6. Induction of Anemia Experimental Design

#### 2.6.1. Ethical Approval

Our laboratory aforementioned above in the affiliation SNAMOPEQ adopts an ethical approval under number L.20. USMBA-SNAMOPEQ 2020-03. The manipulation of rats was respected according standard guidelines approved by our institutional committee on animal care, USMBA, FSDM, Fez, Morocco and the EU Directive 2010/63/EU for animal experiment to avoid and minimize animal suffering and the number of experimental animals [49].

#### 2.6.2. Experimental Protocol

Wistar male rats weighing 140 ± 5 g were used. The animals were acclimated under environmental conditions of 25 °C, 55% humidity and a 12-h light–dark period, within the animal house.

The animals were randomly allocated into three groups of six rats each: Group 1 served as the negative control and received physiological water (10 mL/kg) orally; Group 2 served as the positive control and received phenylhydrazine by intravenous injection for 4 days at a concentration of 10 mg/kg; Group 3 received phenylhydrazine by intravenous injection for 4 days at a concentration of 10 mg/kg and was then treated with apple vinegar (1 mL/day) orally for five weeks.

The treatment duration was chosen according to our previous study [33]. Phenylhydrazine and apple vinegar doses were selected based on the studies of Lee et al. [50] and Ajaykumar et al. [51], respectively.

At the end of the experimental period (5 weeks), the animals were sacrificed after anesthesia. The blood sample and plasma were collected for further biochemical assessments.

### 2.7. Analysis of Hematological Parameters

White blood cells (WBC), hemoglobin concentration (HGB), red blood cells (RBC), hematocrit (HCT), mean capsulated hemoglobin (MCH), mean corpuscular volume (MCV), mean corpuscular hemoglobin concentration (MCHC), and platelets (PLT) were determined using an automatic counter (Sysmex K21, Tokyo, Japan).

### 2.8. Osmotic Fragility Test

An osmotic fragility assay was chosen to examine the relationship between hemolysis and osmolality using a spectrophotometric method as previously described by Swem et al. [52]. Different solutions of NaCl were prepared (0.9%, 0.6%, 0.5%, 0.4%, 0.3% and 0%). A sample of 0.1 mL of suspended red blood cells was mixed with different concentrations of NaCl and then mixed and centrifuged for 15 min at 1500 rpm. The absorbance of the supernatant of each tube was measured at 546 nm using a spectrophotometer (UV–visible spectrophotometer UV-1650PC). Percentage hemolysis was determined using the calibration curve.

### 2.9. Statistical Analysis

In each group, the findings were represented as the mean ± standard deviation (SD) of the variable readings. One-way ANOVA and Tukey post hoc tests were used to compare the groups statistically.

## 3. Results

### 3.1. Phytochemical Quantification and Antioxidant Activity of Apple Vinegar

**Figure 1:**
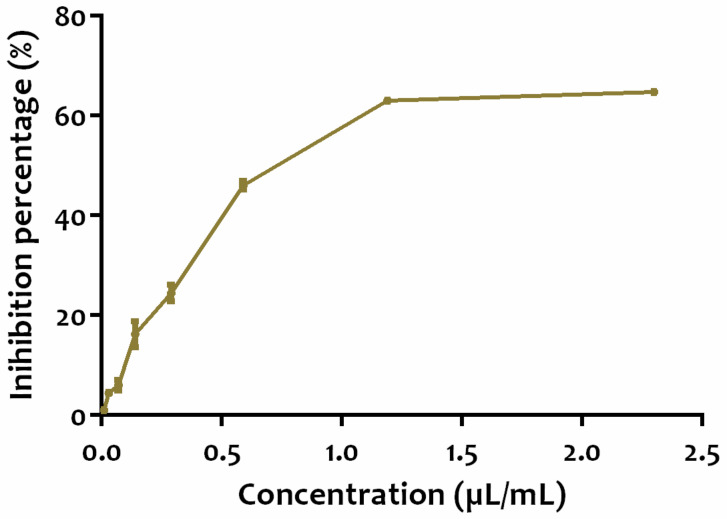
Inhibition percentage of free radical scavenging DPPH of apple vinegar.

The phenolic and flavonoid contents and antioxidant activity of apple vinegar are presented in Table 1. Polyphenolic and flavonoid contents were 90 ± 1.66 mg GAE/100 mL and 7.29 ± 0.23 mg QE/100 mL, respectively. In addition, the total antioxidant capacity of our sample was 4.22 ± 0.18 mg AAE/100 mL, while the ability to scavenge free radical DPPH was 0.49 ± 0.004 µL/mL. Figure 1 displays the evolution of inhibition percentage depending on the concentration of apple vinegar.

### 3.2. The Mineral Content of Apple Vinegar

The mineral composition of apple vinegar is presented in Table 2. The most abundant mineral element was potassium, with a value of 37.87 ± 1.5 mg/L, while iron presented the weakest value with 0.09 ± 0.01 mg/L. Importantly, our sample was free of heavy metals.

### 3.3. Phenolic Profile of Apple Vinegar

The apple vinegar sample included 32 polyphenolic chemicals, all of which were identified and measured (Table 3). As presented in Table 3, the major phenolic components detected were trans-ferrulic acid (43.92%), ferrulic acid (40.15%), sinnapic acid (3.88%), arbutin (3.73%), apigenin (2.53%) and trans-cinammic acid (1.83%). Other phenolic compounds were detected in low concentrations, including oleochantal, hydroxytyrosol, oleuropein, hesperetin, trimethoxyflavone, amentoflavone, quercetin-3-*O*-glucoside, quercetin-3-*O*-glucoronic acid, quercetin-3-*O*.hexose deoxyhexose, isorhamnetin-3-I rutinoside, isorhamneti-7-*O* pentose/luteoilin-7-*O* glucoside, kaempferol-3-*O*-glucuronic acid, kaempferol-3-*O*-hexose deohyhexose, protocathecoic acid, vanillic acid, syringic acid, gentisic acid, chlorogenic acid, cathechin/epicathechin, gallocathechin/epigallocathechingallate, gallocathechin/epigallocathechin, procianidin, myricetin, kampeferol, rutin and narigin. However, cathechin gallate, caffeic acid, *p*-hydrocybenzoic/salicylic acid, tyrosol, kaempferol-3-*O*-glucose, luteolin, ursolic acid, rosmarinic acid, *p*-coumaric acid, quercetin, gallic acid and syringic acid were not detected in apple cider vinegar (Table 3).

### 3.4. The Effect of Apple Vinegar on Different Hematological Markers

**Figure 2:**
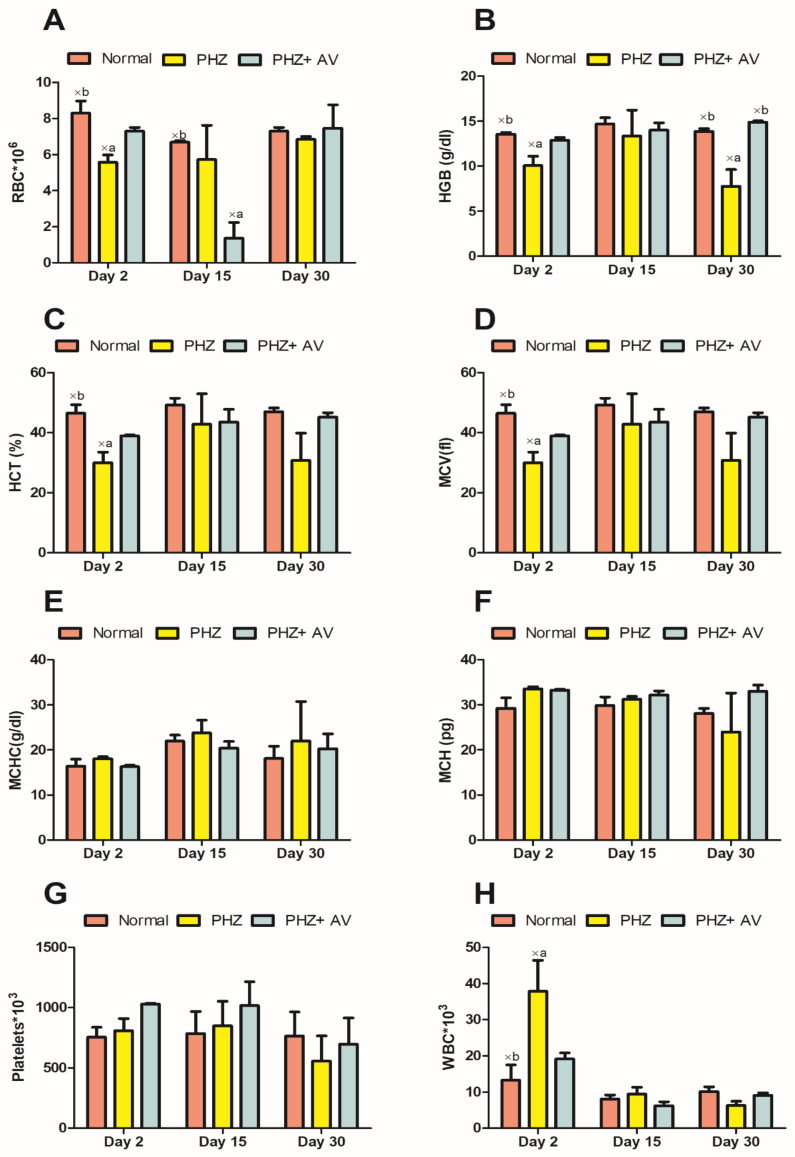
Effect of apple vinegar on hematological indices: **A**: effect of the interventions on red blood cells (RBC); **B**: effect of the interventions on hemoglobin (HGB); **C**: effect of the interventions on hematocrit (HCT); **D**: effect of the interventions on mean corpuscular volume (MCV); **E**: effect of the interventions on mean corpuscular hemoglobin concentration (MCHC); **F**: effect of the interventions on mean capsulated hemoglobin (MCH); **G**: effect of the interventions on platelets; **H**: effect of the interventions on white blood cells (WBC); ×**a**: comparison between normal and the two other groups; ×**b**: comparison between PHZ group and the two other groups (significance *p* < 0.05).

Figure 2 presents the effect of apple vinegar on different hematological markers. The significantly weakest values of RBC, HGB and HCT were registered in the experimental group (PHZ) as compared to the normal group (*p* < 0.05). The results obtained from the current study revealed that rats treated with apple vinegar (1 mL/kg) and PHZ (mg) simultaneously showed an increase in RBC, HGB and HCT values as compared to the positive group. Concerning the platelets level, phenylhydrazine induced an increase in platelets level during the 15 days of the experiment. The level of platelets then decreased on the last day of the experiment. The treatment of rats with apple vinegar ameliorates the level changes of the studied parameters, which were induced by PHZ. The immune cell count revealed that the administration of PHZ increased WCB levels, while apple vinegar gradually normalized the changes induced by PHZ after 30 days of treatment.

### 3.5. The Effect of Apple Vinegar Pretreatment on Erythrocyte Osmotic Fragility of Phenylhydrazine-Poisoned Rats

**Figure 3:**
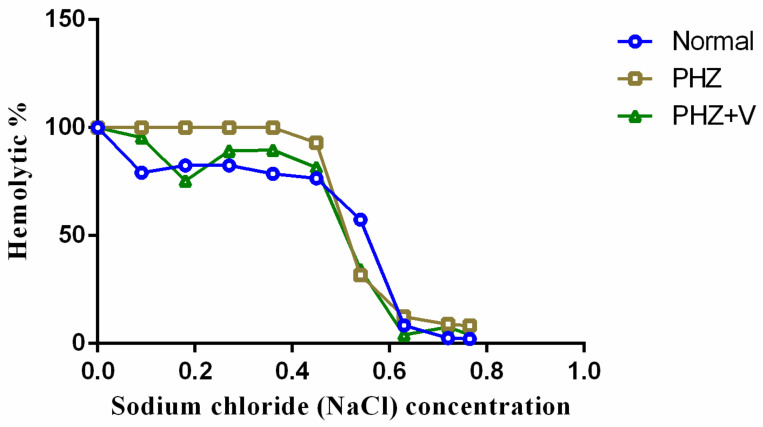
Percentage erythrocyte osmotic fragility of phenylhydrazine-treated and normal rats.

The percentage of hemolysis was dose dependent, as observed in the apple vinegar-treated groups. The obtained results of the osmotic fragility test are summarized in Figure 3. In a 0.6 % sodium chloride dose, hemolysis percentages of 4.10 ± 0.01% and 8.47 ± 0.12% were shown to be lower when apple vinegar was administrated simultaneously with phenylhydrazine-treated and normal rats, respectively, when compared to the phenylhydrazine-poisoned group, with 12.41%. The hemolysis percentage increased depending on the sodium chloride dose, from 0.5% to 0% of NaCl, in all groups.

## 4. Discussion

Apple vinegar has been used since ancient times in folk medicine to prevent and treat different ailments such as diabetes, hyperlipidemia and oxidative stress [33,53,54,55,56,57,58]. The results of the quantification of phenolic and flavonoid contents revealed that apple vinegar contains high concentrations of these molecules, which is in line with previous studies [36,59]. Additionally, apple vinegar exhibited good antioxidant activity, meaning it could protect and prevent organ injuries induced by toxic agents.

It can be clearly seen in the Results section that the administration of apple vinegar ameliorated the parameter changes induced by the injection of phenylhydrazine. It has been reported that the administration of phenylhydrazine enhances red blood cell destruction, which leads to the onset of hemolytic anemia [10,60,61]. Additionally, it increases the production process of reactive oxygen species [11].

Apple-derived products such as apple vinegar have shown tremendous abilities. Omar et al. stated that one of the beneficial properties of apple vinegar is the protection against nicotine-induced oxidative stress in rats and the prevention of liver histological damage [42]. Apple vinegar contains phytochemicals that possess antioxidant properties such as polyphenols and flavonoids [43]. Antioxidant substances proved their ability to maintain the erythrocyte membrane in stress conditions reducing methemoglobin [62,63].

In our recently published review article, we summarized different quality characteristics, phytochemical content and several biological functions of apple vinegar, and we stated that the diverse compounds of fruit vinegars are highly associated with its functional properties and command different pathways to exert antihyperglycemic, antihyperlipidemic and anti-inflammatory effects [37]. This is the first study designed to evaluate the antianemic impact of apple vinegar that advises adding this property to the long list of its biological activities.

The use of vinegar started early. Khan et al. recently described a diet therapy involving eating meats dressed with vinegar or acidic syrup in order to counteract anemia and to empower liver function [64]. Additionally, apple vinegar has been documented to potentiate the intestinal absorption of iron [65]. Citric acid is an organic acid present in apple vinegar, which has been proven to stimulate iron absorption in iron-deficient rats [66]. The bioavailability of iron could be the reason for the marked increase in hemoglobin, unregistered in the current work. It has been proven that iron availability modulates myeloid cell differentiation [67], and its homeostasis adjusts osteoclast development [68]. A mineral analysis of our sample revealed the presence of calcium and magnesium, which modulate the proliferation and differentiation of hematopoietic precursors [69] and regulate erythrocyte biochemical functions [70]. Apple vinegar contains different mineral elements that play a pivotal role in blood cell production and could constitute a good tool for ameliorating mineral deficiency. Furthermore, potassium plays a crucial role in the stability of erythrocytes, as it determines their water and cation contents [71], while its deficiency is the main reason for red blood cells aging [72]. Zinc is involved in the erythropoiesis process as a signal [73], and its deficiency may induce thalassemia and erythrocyte fragility [74].

Phenyl hydrazine destroys red blood cells through the overproduction of ROS, which denaturalize hemoglobin, membrane phospholipids and key enzymes implicated in erythrocyte metabolism [75,76]. In India, ayurvedic formulations were used to treat anemia, such as *Raktavardhak kadha* (RK), which contained 13 polyherbal extracts. RK was proven to be capable of counteracting the deleterious effects of phenylhydrazine-induced anemia in rats [77]. The same authors evoked that RK treatment restored all changes induced by PHZ administration on blood markers and histomorphological tissues, thanks to its complex phytocomposition including flavonoids and phenols [77].

Apple vinegar contains numerous biologically active components such as gallic acid, chlorogenic acid, caffeic acid and catechin [43]. The presence of several bioactive compounds in apple vinegar are effective in inhibiting the installation of different illnesses including diabetes and cancer [78,79]. However, no attention has been paid examining the ability of apple vinegar to prevent phenylhydrazine-induced hemolytic anemia.

Phenolic compounds exhibit a good antioxidant activity, and it has been proved that these antioxidant agents have an antianemic effect, which could correct blood disorders [60]. A study indicated that apple vinegar was rich in antioxidant substances including chlorogenic acid, gallic acid, p-coumaric acid, caffeic acid, catechin, epicatechin gallate and phlorizin [43,80].

In fact, trans-ferulic acid, ferulic acid, sinapic acid, arbutin, trans-cinnamic acid and apigenin were the most predominant polyphenolic components identified in our apple vinegar sample. Mounting evidence has proved that ferulic acid possesses a cytoprotective effect and hinders gene expression controlling cell apoptosis due to its effect on electron donation, which enhances its excellent antioxidant ability [81,82]. In addition, trans-ferulic acid counteracts cytotoxicity induced by chemotherapy drugs and defends oxidative damage induced by reactive oxygen species. Furthermore, trans-ferulic acid ameliorates antioxidant enzyme levels [83]. Arbutin and apigenin were multifunctional molecules used in phytotherapy and phytocosmetics [84].

Ferulic acid is extensively distributed throughout the plant world, particularly in the Ranunculaceae and Gramineae families [85]. Ferulic acid is frequently associated with lignin and polysaccharide to constitute plant cell walls, although it is seldom seen in its pure form. Ferulic acid is a multifunctional bioactive molecule and its derivatives present diverse biological functions. It functions as both an antioxidant and an anti-inflammatory agent. To resist oxidative damage and minimize inflammatory reactions, it may eliminate abundant ROS or control their production process [86]. It seems to protect against renal [87,88] and cardiovascular diseases [89]. Ferulic acid may also prevent thrombosis by inhibiting platelet aggregation and the production of thromboxane-like compounds [90], inhibiting hepatic cholesterol synthesis in lower blood lipid [91], and preventing coronary heart disease [92] and atherosclerosis [85], among other things. Phenolic compounds, especially flavonoids, appear to be the main substances responsible for bone marrow protection against the deleterious effects of phenylhydrazine [77]. In a study conducted by Zheng et al., the results showed that flavonoids can induce the expression of erythropoietin (EPO). Therefore, it could be used in the improvement of hematopoietic functions. It was found that flavonoids increase the expression of the erythropoietin gene via two distinct pathways, the first being the increase in the expression of hypoxia-inducible factor-1α (HIF-1 α), the key regulator of EPO expression, and the second being the reduction in the degradation of HIF-1 α, and HIF-1 α-OH [93].

Bioactive ingredients can play a crucial role in the elimination of ROS and can neutralize heavy metals, thereby maintaining homeostasis. A study published by [32] revealed that apple vinegar enhanced the antioxidant defense system, which could decrease enzyme leakage into plasma. The same findings were evoked by [41]. In relation to our previous studies, recently, we proved that apple vinegar exhibited an important antioxidant effect in vivo against hydrogen peroxide at the dose of 10 mL/kg b.w during 22 days of treatment by reducing liver and kidney damage and decreasing hepatic enzymes and creatinine [38]. These protective effects were related to the phenolic and flavonoid contents of apple vinegar [38].

The synergistic interaction between bioactive ingredients of apple vinegar provides its ability to counteract phenylhydrazine-induced hemolytic anemia.

## 5. Conclusions

This is the first report designed to evaluate the antianemic potency of apple vinegar. Besides all other health benefits presented by apple vinegar reported previously, the data presented in this paper support its further consumption and use for other health benefits, especially for hematological problems and anemia. The promising antioxidant results shown in this study support the use of apple vinegar as a diet supplement in the prevention and treatment of oxidative stress-related diseases such as diabetes, inflammation and other pathologies.

## Figures and Tables

**Table 1:** Total polyphenols and flavonoids content and total antioxidant capacity of apple vinegar.

	TPC mg GAE/100mL	TFC mg QE/100mL	TAC mg AAE/100mL	IC_50_% DPPH µL/mL
Apple vinegar	90 ± 1.66	7.29 ± 0.23	4.22 ± 0.18	0.49 ± 0.004

**Table 2:** Mineral composition of apple vinegar.

	Minerals Content (mg/L)					
Apple vinegar	K	Na	Mg	Ca	Fe	P
	37.87 ± 1.5	6.12 ± 0.8	3.72 ± 0.3	3.21 ± 0.1	0.11 ± 0.01	0.09 ± 0.13
	Zn	Pb	Cu	Cr	Cd	
	0.24 ± 0.01	ND	ND	ND	ND	

**Table 3:** Phenolic compounds and quantification of apple vinegar.

Phenolic Compounds in Apple Vinegar (%)					
Syringic acid	Gallic acid	Kaempferol	Rutin	Oleochantal	Hydroxytyrosol
ND	ND	0.192	0.125	0.132	0.019
Transferulic acid	Oleuropein	Hesperetin	Trimethoxyflavone	Arbutin	Rosmarinic acid
43.921	0.004	0.015	0.102	3.736	ND
Ursolic acid	Apigenin	Amentoflavone	Luteolin	Quercetin-3-*O*-glucoside	Quercetin-3-*O*- glucuronic acid
ND	2.539	0.047	ND	0.021	0.044
Kaempferol-3-*O*-glucose	Quercetin-3-*O*-hexose deoxyhexose	Isorhamnetin- 3-*O* Rutinoside	Isorhamnetin-7-*O*- Pentose / luteoilin 7-*O*-glucoside	Kaempferol-3-*O*-glucuronic acid	Narigin
ND	0.031	0.016	0.171	0.102	0.104
Kaempferol-3-*O*-hexose deohyhexose	Tyrosol	Protocathecoic acid	Vanillic acid	Syringic acid	*p*-hydroxybenzoic\ salicilic acid
0.054	ND	0.238	0.193 ± 0.002	0.953 ± 0.013	ND
Gentisic acid	Caffeic acid	Ferulic acid	Sinapic acid	Trans-cinnamic acid	Chlorogenic acid
0.153	ND	40.155	3.887	1.835	0.372
Cathechin\ epicathechin	Gallocathechin\ epigallocathechin gallate	Gallocathechin\ epigallocathechin	Cathechin gallate	Procianidin	Myricetin
0.213	0.255	0.128	ND	0.093	0.146

ND: Not determined.

## Data Availability

Data are available upon request.

## Supplementary Materials

The following are available online at https://www.mdpi.com/article/10.3390/life12020239/s1, File S1: LC-MS/MS analysis Data.
